# Temporal Changes in Invasive Group B *Streptococcus* Serotypes: Implications for Vaccine Development

**DOI:** 10.1371/journal.pone.0169101

**Published:** 2016-12-30

**Authors:** Ziyaad Dangor, Clare L. Cutland, Alane Izu, Gaurav Kwatra, Siobhan Trenor, Sanjay G. Lala, Shabir A. Madhi

**Affiliations:** 1 Medical Research Council: Respiratory and Meningeal Pathogens Research Unit, Faculty of Health Sciences, University of the Witwatersrand, Johannesburg, South Africa; 2 Department of Science and Technology/National Research Foundation: Vaccine Preventable Diseases, Faculty of Health Sciences, University of the Witwatersrand, Johannesburg, South Africa; 3 Department of Paediatrics & Child Health, Faculty of Health Sciences, University of the Witwatersrand, Johannesburg, South Africa; 4 National Institute for Communicable Diseases: a division of National Health Laboratory Service, Johannesburg, South Africa; New York University School of Medicine, UNITED STATES

## Abstract

**Introduction:**

There is a paucity of longitudinal data on the serotype-specific burden of invasive group B *Streptococcus* (GBS) disease from low-middle income countries, which could inform selection of vaccine epitopes.

**Methods:**

From 2005 to 2014, infants less than 90 days of age with invasive GBS disease were identified through sentinel laboratory and hospital admission surveillance at Chris Hani Baragwanath Academic Hospital in Soweto, South Africa.

**Results:**

We identified 820 cases of invasive GBS disease, including 55% among newborns <7 days age (i.e. early-onset disease; EOD). The overall incidence (per 1,000 live births) of invasive GBS disease was 2.59 (95% CI: 2.42–2.77), including 1.41 (95% CI: 1.28–1.55) for EOD and 1.18 (95% CI: 1.06–1.30) in infants 7–89 days age (late-onset disease). Year-on-year, from 2005 to 2014, we observed a 9.4% increase in incidence of serotype Ia invasive disease (RR: 1.09; 95% CI: 1.04–1.15; p<0.001), and a 7.4% decline in serotype III invasive disease (RR: 0.93; 95% CI: 0.90–0.96; p<0.001). Overall, serotypes Ia (28.2%), III (55.4%) and V (7.9%) were the commonest disease causing serotypes.

**Conclusions:**

The incidence of invasive GBS disease has remained persistently high in our setting, with some changes in serotype distribution, albeit mainly involving the same group of dominant serotypes.

## Introduction

The incidence of invasive group B *Streptococcus* (GBS) disease in infants less than 90 days-of-age varies within and between countries [1, 2]. Although intrapartum antibiotic prophylaxis (IAP) to at-risk or GBS-colonized pregnant women has successfully reduced the burden of early-onset disease (<7 days age; EOD) [3], GBS remains the commonest cause of sepsis and meningitis in young infants in high-income countries [4–6]. In many low and middle income countries (LMIC), laboratory resource constrains, difficulty with accessing health care facilities, empiric antibiotic treatment prior to hospital referral and differing thresholds for investigating bacterial disease might hamper identification of pathogens associated with severe bacterial infections, including GBS [7–9]. This was corroborated recently in South Africa, where the incidence of invasive GBS across the nine provinces ranged from 0.02 to 2.28 per 1,000 live births, with the highest incidence reported from the two most resourced provinces [10]. Consequently, active sentinel surveillance is likely required to fully characterize the burden of invasive GBS disease in LMIC. Such surveillance could also inform whether there are temporal changes in serotype distribution, which could inform the development of polysaccharide-based vaccines aimed at immunization of pregnant women.

Screening for GBS colonization in pregnant women is not routinely undertaken in the public health sector in South Africa, however, IAP is recommended during labor to pregnant women with risk factors for neonatal sepsis, including the presence of spontaneous preterm labor, amniotic membranes rupture ≥18hours prior to birth or the presence of intrapartum fever. The implementation of this risk-based strategy is, however, not optimal with only 11–26% of women with risk-factors receive IAP [11, 12].

The objective of this study was to investigate the trends in incidence of serotype-specific invasive GBS disease over a decade in a low-income South African setting among black-Africans.

## Materials and Methods

Since 2005, clinical and laboratory surveillance has been ongoing at Chris Hani Baragwanath Academic Hospital (CHBAH) in Soweto, Johannesburg. Approximately 22,000 live births occur at CHBAH annually and a further 10,000 live births occur at the seven midwife-operated obstetric units (MOU’s) within and near Soweto [13]. Until 2014, when a smaller district hospital opened, CHBAH was the only public hospital serving Soweto. During the period of observation for this study, newborns and infants with possible severe bacterial infection would likely have been referred to CHBAH. Although South Africa is classified as an upper middle income country by the World Bank, the country has a high GINI co-efficient (0.660 to 0.696), and the populace of Soweto are largely low-income earners: 40% of the population has an income of <US$2 per day and the unemployment prevalence is 53% [14, 15]. Public health care is offered at no fee to pregnant women and children <6 years of age in South Africa. Overall, only 16% of families have private medical insurance, with this figure being even lower in Soweto (<10%) [16].

Clinicians at CHBAH have a low threshold to hospitalize and investigate young infants with suspected sepsis or meningitis, and blood and cerebrospinal fluid (CSF) cultures are routinely undertaken as per standardized guidelines. In this analysis, we included all infants less than 90 days of age admitted at CHBAH from whom GBS was cultured on blood or CSF, or in whom GBS was identified on CSF by the latex agglutination test. Meningitis was defined as CSF culture or latex agglutination positivity for GBS, or a positive blood culture in a child in whom the CSF cell-count was suggestive of meningitis (i.e. pleocytosis ≥20 cells/mm^3^ for neonates or ≥10 cells/mm^3^ for infants between 29 and 89 days of age). Early-onset disease was defined as disease occurring within 7 days of age and late-onset disease (LOD) as illness occurring between 7–89 days of age.

Maternal HIV prevalence in this province has remained at approximately 30% since 2001 [17]. The recommendations for prevention of mother to child transmission and HIV-management of HIV-infected pregnant women has evolved over the past decade in South Africa [18, 19]. Briefly, since 2001, pregnant women with CD4+ lymphocyte counts >200 cells/mm3 and later (2008) >350 cells/mm3 and WHO stage 1 and 2 received single therapy with zidovudine or Nevirapine; whereas those with CD4+ lymphocyte counts <350 cells/mm3 or WHO stage 3 or 4 were initiated on triple antiretroviral therapy (ART). From April 2013, all pregnant women irrespective of CD4+ lymphocyte counts were initiated on ART [19].

### Laboratory methods

Standard laboratory methods for the identification of GBS on culture have been described [12]. Briefly, GBS from blood was isolated using the Bact/Alert microbial system (Organon Teknika, Durham, NC), plated on blood or chocolate agar. On CSF samples, gram-staining was done and samples plated onto blood or chocolate agar plates were inoculated into an enrichment broth (Brain Heart Infusion, Diagnostics Media Production; South Africa). A GBS antigen agglutination test (Wellcogen Bacterial Antigen Kit, Remel, UK) was also undertaken if the CSF culture was negative, but the CSF cyto-chemistry was suggestive of bacterial meningitis. Positive GBS isolates were archived at -70 degrees celsius at the Respiratory and Meningeal Pathogens Research Unit. Serotyping (Ia, Ib, II–IX) methods were consistently performed over the study period using latex agglutination (Statens Serum Institute, SSI, Sweden) [20, 21]. Twelve (1.9%) non-typeable isolates were further characterized using single-plex PCR and primers as described (S1 Table) [22, 23].

### Statistical analysis

Demographic characteristics were compared between infants with EOD and LOD using the Chi-squared or Mann Whitney test. The incidence (per 1,000 live births) was calculated as the number of hospitalized infants with invasive GBS disease at CHBAH by the total number of live births that occurred at CHBAH and the seven MOU’s within and near Soweto, where <1% of women deliver at home. The incidence was stratified by EOD and LOD, as well as by maternal HIV infection status. From this setting, we have previously published on the incidence and serotype distribution for the period 2005–2008 and over a single year from 2012–2013 [12, 24]. In this paper, we explore temporal trends of incidence for invasive GBS disease, including serotype-specific incidence over a 10-year period (S1 Dataset).

We evaluated the presence of a non-zero linear or quadratic trend in monthly incidence rates using least squares regression. A non-linear model was used to model the monthly incidence (per 1,000 live births) of GBS during the study period. Because EOD and LOD had smaller monthly incidence rates, we fitted the INGARCH model to this data [25]. A 5-term moving average was used to highlight trends in HIV-exposed and unexposed infants, and for serotype distributions. A 5-term moving average is the average of the two-previous, index and following two month incidence (per 1,000 live births) plotted against time and is used to smooth out short-term variations when examining time-series data. For example, the 5-term moving average for March 2005 would be the average of the incidence rates in January 2005 to May 2005. Year-on-year relative risk (RR) was reported for changes in serotype trends. Serotype data was unavailable on 185 (22.6%) of cases; 47 (56.0%) in 2005, 27 (35.1%) in 2006, 27 (34.2%) in 2007, 21 (23.1%) in 2008, 27 (30.0%) in 2009, 13 (16.5%) in 2010, 13 (16.9%) in 2011, 8 (9.4%) in 2012 and 2 (2.3%) in 2013. For missing serotype data, we extrapolated the number of serotype-specific cases based on the available data.

Data were analyzed using STATA version 13.1 (College Station, Texas, USA), SAS version 9.4 (Cary, NC, USA) and R version 3.1.2 (Vienna, Austria). The study was approved by the University of Witwatersrand Human Research Ethics Committee (HREC number: M031007/ M10367). Informed, written consent was obtained from mothers of infants. For infants that demised or were discharged prior to consenting, permission to retrospectively review records and obtain the laboratory isolate was granted by the ethics committee.

## Results

Over the 10-year period, 820 cases of invasive GBS disease were identified, of which 447 (54.5%) were EOD and 373 (45.5%) were LOD (Table 1, S2 Table). Infants with invasive GBS disease had a median gestational age of 38 weeks (IQR: 35–40 weeks); 30.8% of infants were <37 weeks and 18.4% of infants were <34 weeks. Infants with EOD compared to LOD cases were more likely to be born prematurely (37.5% vs 22.6%, respectively; p<0.001) or have a birth weight <2500 grams (37.0% vs 28.8%, respectively; p = 0.013). The overall case fatality ratio was 15.7% (128 of 820), which was similar between EOD (15.4%) and LOD (16.0%) cases. Infants with LOD were 8.18 (95% CI: 5.78–11.60) fold more likely to present as meningitis (58.2%) than infants with EOD (14.5%; p<0.001). Forty two percent (n = 346/820) of cases were HIV-exposed, whilst the HIV-exposure was unknown in 51 (6.2%) cases. The odds of an infant with LOD being HIV-exposed (54.4%) was 2.67 (95% CI: 1.97–3.61; p<0.001) fold higher than for infants with EOD (32.0%).

**Table 1:** Demographic characteristic of infants with invasive Group B streptococcus disease.

	Overalln (%)	EOD^a^n (%)	LOD^b^n (%)	p-value^c^
**Total number of cases**	820	447	373	
**HIV-status**				
HIV-unexposed	423 (51.6)	276 (61.7)	147 (39.4)	<0.001
HIV-exposed	346 (42.2)	143 (32.0)	203 (54.4)	
HIV-unknown	51 (6.2)	28 (6.3)	23 (6.2)	
**Gender**				
Male	435 (53.1)	238 (53.2)	197 (52.8)	0.903
**Gestation**				
Median(IQR^d^)	38 (35–40)	38 (34–39)	38 (37–40)	<0.001
≥37 weeks	556 (69.2)	276 (62.5)	280 (77.4)	<0.001
<37 - ≥34 weeks	100 (12.4)	62 (14.0)	38 (10.5)	
<34 weeks	148 (18.4)	104 (23.5)	44 (12.1)	
**Birth Weight**				
Median(IQR)	2800 (2188–3200)	2805 (1965–3230)	2800 (2400–3140)	0.383
≥2500 grams	539 (66.7)	279 (63.0)	260 (71.2)	0.013
1500–2499 grams	178 (22.0)	98 (22.1)	80 (21.9)	
1000–1499 grams	65 (8.1)	43 (9.7)	22 (6.0)	
≤999 grams	26 (3.2)	23 (5.2)	3 (0.9)	
**Outcome**				
Discharged	688 (84.3)	378 (84.6)	310 (84.0)	0.829
Demised	128 (15.7)	69 (15.4)	59 (16.0)	
**Culture site**				
Blood only	538 (65.6)	382 (85.5)	156 (41.8)	<0.001
CSF^e^ only	83 (10.1)	11 (2.5)	72 (19.3)	
Blood and CSF	133 (16.2)	23 (5.1)	110 (29.5)	
CSF suggestive^f^	66 (8.1)	31 (6.9)	35 (9.4)	

^a^EOD- Early-onset disease

^b^LOD- Late-onset disease

^c^p-value comparing EOD and LOD using Chi-squared or Wilcoxon rank-sum (Mann-Whitney) test

^d^IQR- interquartile range

^e^CSF- Cerebrospinal fluid

^f^CSF suggestive of meningitis was defined as pleocytosis ≥20 cells/mm3 for <28 day-olds and ≥10 cells/mm3 for 29–89 day-olds with no adjustment made for traumatic taps and in the absence of positive CSF culture.

### Trends in incidence of invasive GBS disease

**Fig 1:**
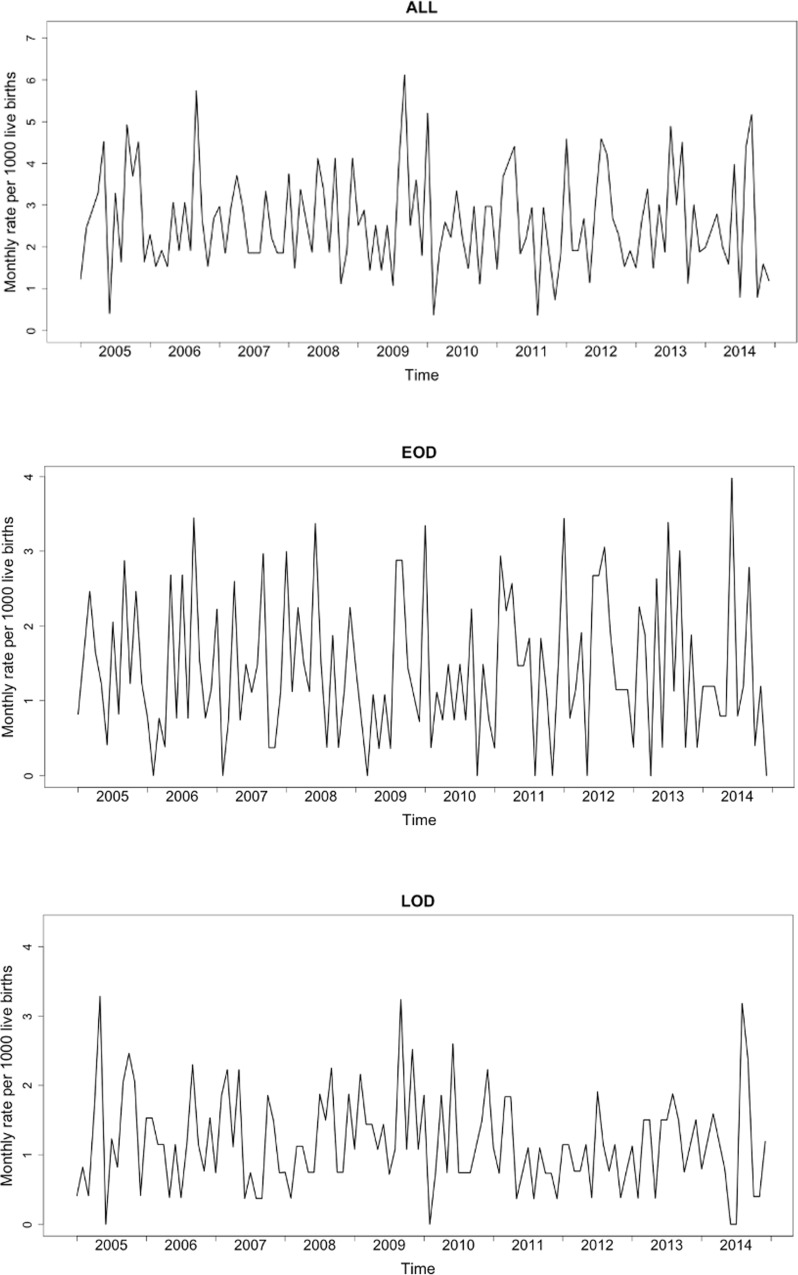
Monthly incidence of invasive GBS disease in infants less than 90 days of age for All (A), early-onset disease (B) and late-onset disease (C)

The overall incidence (per 1,000 live births) of invasive GBS disease over the 10-year period was 2.59 (95% CI: 2.42–2.77); which remained similar throughout the study period ($\chi_{trend}^{2}$ = 0.356, p = 0.551). The incidence for EOD was 1.41 (95% CI: 1.28–1.55; $\chi_{trend}^{2}$ = 0.005, p = 0.945) and 1.18 (95% CI: 1.06–1.30; $\chi_{trend}^{2}$ = 0.922, p = 0.337) for LOD (Fig 1A–1C and S3 Table). No seasonal patterns were observed for the overall, EOD and LOD incidence.

**Fig 2:**
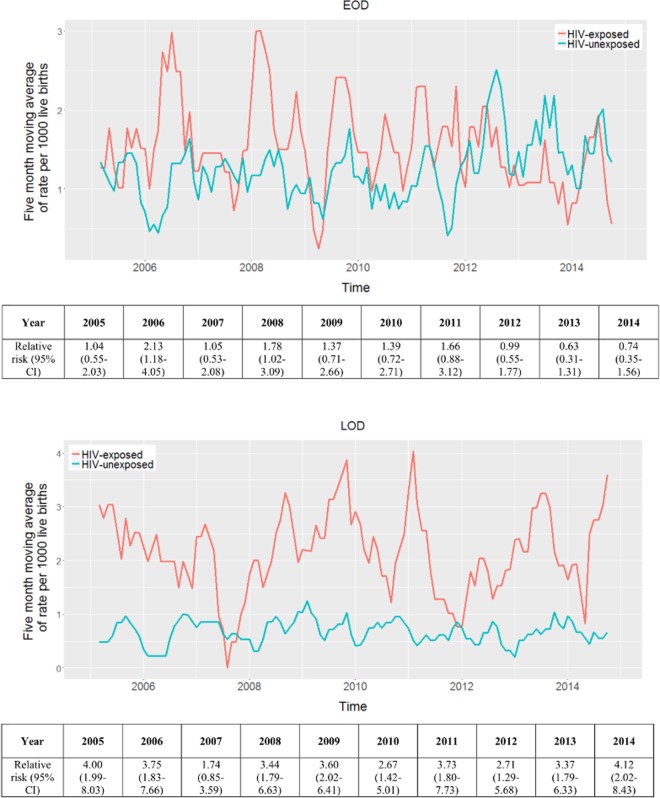
**Five-term moving average of early-onset disease (A) and late-onset disease (B) stratified by HIV-exposure status.** Footnote: Relative risk comparing HIV-exposed and unexposed infants per year.

The overall incidence of invasive GBS disease was 1.92-fold (95% CI: 1.67–2.21; p<0.001) higher in HIV-exposed (3.66; 95% CI: 3.28–4.06) than HIV-unexposed (1.90; 95% CI: 1.73–2.10; p<0.001) infants. HIV-exposed infants had an increased risk for LOD (RR; 3.24; 95% CI: 2.62–4.01; p<0.001), and similarly so for EOD (RR; 1.22; 95% CI: 0.99–1.49; p = 0.057); albeit not significant (Fig 2A and 2B, S3 Table).

### Serotype distribution of cases of invasive GBS disease

Proportionately, the number of invasive GBS disease cases caused by serotype III declined from 70.3% in 2005 to 47.2% in 2014 (p = 0.022; S4 Table, S1 Fig). In contrast, the number of cases caused by serotype Ia increased from 16.2% in 2005 to 44.2% in 2012 (p = 0.002), although there was a non-significant downward trend thereafter by 2014 (25.0%; p = 0.295). The proportion of cases caused by serotype V did not vary significantly during the study period ranging from 8.1% in 2005 to 13.9% in 2014; p = 0.378. Overall, the most prevalent serotypes for EOD were Ia (34.7%), III (41.4%) and V (11.4%), whereas serotypes III (73.8%) and Ia (19.6%) were most prevalent for LOD. Serotype Ib and II accounted for 3.5% and 3.6% of all cases respectively (S4 Table, S1 Fig). Serotypes did not vary significantly between HIV-exposed and -unexposed infants (data not shown).

**Fig 3:**
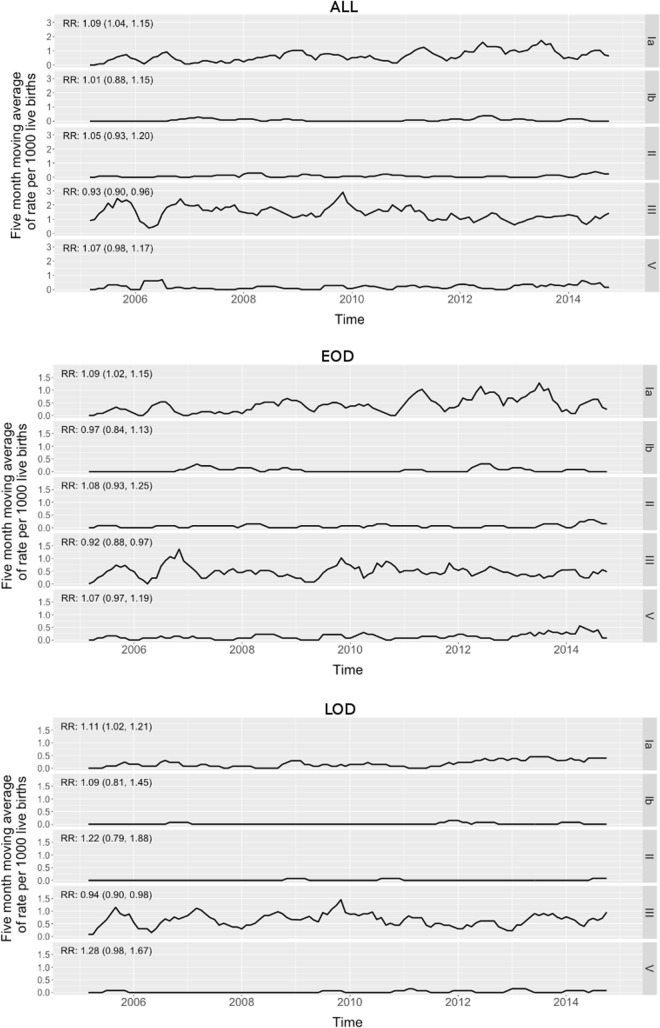
Five-term moving average and year-on-year relative risk (RR; 95% confidence interval) of serotype-specific incidence rates of invasive GBS disease for All (A), early-onset disease (B) and late-onset disease (C).

The 5-term moving averages per 1,000 live births for the five most prevalent serotypes (Ia = 28.2%, Ib = 3.4%, II = 3.6%, III = 55.4%, V = 7.9%) are shown in Fig 3A–3C. There was a 9.4% increase in the incidence (per 1,000 live births) of cases caused by serotype Ia year-on-year (RR: 1.09; 95% CI: 1.04–1.15; p<0.001), which was apparent for EOD (RR: 1.09; 95% CI: 1.02–1.15; p = 0.005) and LOD (RR: 1.11; 95% CI: 1.02–1.21; p = 0.016). In contrast, there was a 7.4% decline in incidence (per 1,000 live births) of cases caused by serotype III year-on-year (RR: 0.93; 95% CI: 0.90–0.96; p<0.001), which was evident for EOD (RR: 0.92; 95% CI: 0.88–0.97; p = 0.002) and LOD (RR: 0.94; 95% CI: 0.90–0.98; p = 0.004). No significant changes in the linear trends were observed for serotypes Ib (p = 0.914), II (p = 0.428) and V (p = 0.125; Fig 3A–3C), albeit the proportion of serotype II and V cases increased to 8.3% and 13.9% in 2014, respectively.

## Discussion

The high incidence of invasive GBS disease in Soweto, South Africa, typical of a low-middle income setting, has remained unchanged over the past decade, and this incidence has remained similar to a previous study from the same site undertaken from 1997–1999 [26]. Our incidence was also comparable to an even earlier report from another South African study among infants born to women of Indian descent undertaken from 1986–1989, where the incidence of invasive GBS disease was 2.65 per 1000 live births [27]. The sustained high incidence of invasive GBS disease reported at our site is seven-fold higher than the national passive-laboratory based surveillance estimates (0.38 per 1,000 live births) [10]. This difference is most likely explained by varying access to health and laboratory services, as well as the clinical thresholds used for investigating invasive GBS disease among infants; with the highest incidence reported from the provinces with the best-developed health infrastructure. In our setting, >99% of births occur at a health facility (the majority of births occur at CHBAH) where, prior to the commencement of antibiotic therapy, blood cultures are routinely obtained from neonates with respiratory distress or birth asphyxia. Consequently 65–95% of EOD cases in this facility are identified on blood culture obtained within a few hours of birth [12, 24, 26], which is similar to observations from USA prior to widespread IAP implementation [28]. These data support that the incidence of invasive GBS disease is likely to be underestimated in LMIC where access to health and laboratory facilities is limited and particularly if newborns are empirically treated with antibiotics prior to blood cultures being undertaken.

Although universal screening of pregnant women for GBS colonization is impractical in our setting, our results strongly suggest that the existing logistical, resource and cost constraints limit the effectiveness of the risk-based IAP strategy which is recommended in South Africa. However, even if a risk-based IAP strategy was more successfully implemented, the overall impact against EOD would be modest because approximately 50% of mothers of newborns with EOD do not have any recognizable underlying risk factors [28]. Furthermore, IAP generally has had no impact on the incidence of LOD (even in well-resourced settings with a negligible HIV burden) [3]. This therefore underscores the need for alternate preventative strategies, such as maternal vaccination to reduce the incidence of invasive GBS disease.

Studies evaluating a trivalent polysaccharide-protein conjugate GBS vaccine have recently been completed in pregnant HIV-infected and -uninfected women [29, 30]. This vaccine, directed against serotypes Ia, Ib and III, could theoretically prevent approximately 79% of invasive GBS disease globally, more especially in developed settings [1]. Our results show that the relative contribution of disease-causing serotypes changed over time in our setting, however, serotypes III and Ia accounted for the majority of invasive GBS disease cases during this period. Our results also highlight the temporal changes in invasive GBS disease caused by non-trivalent vaccine serotypes, especially serotypes II and V. In our setting, this would have inferred lower trivalent vaccine coverage in 2014 (75%) compared to the first five years (89%) of our observation period. Non-vaccine serotypes also contribute to a significant burden of invasive disease in other settings, including the occurrence of serotype IV (2.7%) in the USA [31], and possibly serotype VII (37.1%) in Bangladesh [32]. The reasons for the changing spectrum of GBS serotypes causing disease are unclear; susceptible women may be colonized by different GBS strains, and it is also hypothesized that maternal immunological responses vary in response to established versus recent colonization [33]. Taken together, these results suggest that routine surveillance of disease-causing serotypes should continue and this might guide both polysaccharide-based vaccine development and deployment.

A limitation of our study was that 22.6% (185/820) percent of GBS isolates were unavailable for serotyping because they were discarded by the laboratory prior to retrieval; however, we accounted for this missing data when calculating incidence by disease serotype. Furthermore, our incidence estimates are likely to be a conservative because approximately 3% of neonates in this population demise annually outside a health facility (unpublished data).

In conclusion, the unremitting high burden of invasive GBS disease in this low-middle income setting indicates that an alternative strategy to risk-based IAP is needed for the prevention of invasive GBS disease in infants. Our study demonstrates that disease-causing GBS serotypes vary with time and that successful implementation of a maternal vaccination strategy will depend on continued surveillance of disease-causing serotypes.

## Supporting Information

S1 Dataset Ten-year GBS dataset. (XLSX)

S1 Fig **Serotype distribution of infants with invasive GBS disease; overall (A), early-onset disease (B) and late-onset disease (C).** Serotype data was missing on 47 (56.0%) in 2005, 27 (35.1%) in 2006, 27 (34.2%) in 2007, 21 (23.1%) in 2008, 27 (30.0%) in 2009, 13 (16.5%) in 2010, 13 (16.9%) in 2011, 8 (9.4%) in 2012, 2 (2.3%) in 2013 and nil for 2014.
(DOCX)

S1 Table PCR typing of isolates that were non-typeable by latex agglutination method. (DOCX)

S2 Table Demographic characteristics of infants with invasive Group B streptococcus disease stratified by year. ^a^EOD- Early-onset disease, ^b^LOD- Late-onset disease, ^c^IQR- interquartile range, ^d^CSF- Cerebrospinal fluid, ^e^CSF suggestive of meningitis was defined as pleocytosis ≥20 cells/mm3 for <28 day-olds and ≥10 cells/mm3 for 29–89 day-olds with no adjustment made for traumatic taps and in the absence of positive CSF culture. *Data from 2005 to 2008 and from 2012 to 2013 have been previously reported [12, 24].
(DOCX)

S3 Table Yearly incidence (95% Confidence interval) of infants with invasive Group B streptococcus disease. ^a^EOD- Early-onset disease, ^b^LOD- Late-onset disease, ^c^relative risk and 95% confidence interval comparing HIV-exposed and unexposed infants.
(DOCX)

S4 Table Serotype proportion (95% confidence interval) of infants with invasive GBS disease. Serotype data was missing on 47 (56.0%) in 2005, 27 (35.1%) in 2006, 27 (34.2%) in 2007, 21 (23.1%) in 2008, 27 (30.0%) in 2009, 13 (16.5%) in 2010, 13 (16.9%) in 2011, 8 (9.4%) in 2012, 2 (2.3%) in 2013 and nil for 2014.
(DOCX)
