# Supplementary material for: Temporal Changes in Invasive Group B Streptococcus Serotypes: Implications for Vaccine Development
Source: PLoS One. 2016 Dec 30;11(12):e0169101. doi: 10.1371/journal.pone.0169101 (PMC5201280; doi:10.1371/journal.pone.0169101)
Supplement: S1 Fig — Serotype distribution of infants with invasive GBS disease; overall (A), early-onset disease (B) and late-onset disease (C). Serotype data was missing on 47 (56.0%) in 2005, 27 (35.1%) in 2006, 27 (34.2%) in 2007, 21 (23.1%) in 2008, 27 (30.0%) in 2009, 13 (16.5%) in 2010, 13 (16.9%) in 2011, 8 (9.4%) in 2012, 2 (2.3%) in 2013 and nil for 2014. (DOCX) [file pone.0169101.s002.docx]

S1a Fig:

Serotype data was missing on 47 (56.0%) in 2005, 27 (35.1%) in 2006, 27 (34.2%) in 2007, 21 (23.1%) in 2008, 27 (30.0%) in 2009, 13 (16.5%) in 2010, 13 (16.9%) in 2011, 8 (9.4%) in 2012, 2 (2.3%) in 2013 and nil for 2014.

S1b Fig:

S1c Fig:
