# Supplementary material for: Temporal Changes in Invasive Group B Streptococcus Serotypes: Implications for Vaccine Development
Source: PLoS One. 2016 Dec 30;11(12):e0169101. doi: 10.1371/journal.pone.0169101 (PMC5201280; doi:10.1371/journal.pone.0169101)
Supplement: S1 Table — (DOCX) [file pone.0169101.s003.docx]

S1 Table: PCR typing of isolates that were non-typeable by latex agglutination method

| PCR-type | n=12 (%) |
| --- | --- |
| Ia | 2 (16.7) |
| Ib | 2 (16.7) |
| II | 2 (16.7) |
| III | 3 (25.0) |
| IV | 1 (8.3) |
| V | 2 (16.7) |
