# Supplementary material for: Temporal Changes in Invasive Group B Streptococcus Serotypes: Implications for Vaccine Development
Source: PLoS One. 2016 Dec 30;11(12):e0169101. doi: 10.1371/journal.pone.0169101 (PMC5201280; doi:10.1371/journal.pone.0169101)
Supplement: S2 Table — aEOD- Early-onset disease, bLOD- Late-onset disease, cIQR- interquartile range, dCSF- Cerebrospinal fluid, eCSF suggestive of meningitis was defined as pleocytosis ≥20 cells/mm3 for <28 day-olds and ≥10 cells/mm3 for 29–89 day-olds with no adjustment made for traumatic taps and in the absence of positive CSF culture. *Data from 2005 to 2008 and from 2012 to 2013 have been previously reported [12, 24]. (DOCX) [file pone.0169101.s004.docx]

S2Table: Demographic characteristics of infants with invasive Group B streptococcus disease stratified by year

| **Year** | **2005** | **2006** | **2007** | **2008** | **2009** | **2010** | **2011** | **2012** | **2013** | **2014** |
| --- | --- | --- | --- | --- | --- | --- | --- | --- | --- | --- |
|  | n (%) | n (%) | n (%) | n (%) | n (%) | n (%) | n (%) | n (%) | n (%) | n (%) |
| **Total cases** | 84 | 77 | 79 | 91 | 90 | 79 | 77 | 85 | 86 | 72 |
| **EOD^a^** | 46 (54.8) | 40 (52.0) | 41 (51.9) | 54 (59.3) | 39 (43.3) | 39 (49.4) | 47 (61.0) | 55 (64.7) | 47 (54.7) | 39 (54.2) |
| **LOD^b^** | 38 (45.2) | 37 (48.0) | 38 (48.1) | 37 (40.7) | 51 (56.7) | 40 (50.6) | 30 (39.0) | 30 (35.3) | 39 (45.3) | 33 (45.8) |
| **HIV-status** |  |  |  |  |  |  |  |  |  |  |
| **HIV-unexposed** | 38 (45.2) | 31 (40.3) | 43 (54.4) | 44 (48.4) | 43 (47.8) | 41 (51.9) | 36 (46.8) | 51 (60.0) | 54 (62.8) | 42 (58.3) |
| **HIV-exposed** | 36 (42.9) | 38 (49.4) | 25 (31.6) | 44 (48.4) | 43 (47.8) | 35 (44.3) | 34 (44.2) | 31 (36.5) | 31 (36.0) | 29 (40.3) |
| **HIV-unknown** | 10 (11.9) | 8 (10.4) | 11 (13.9) | 3 (3.3) | 4 (4.4) | 3 (3.8) | 7 (9.1) | 3 (3.5) | 1 (1.2) | 1 (1.4) |
| **Gender** |  |  |  |  |  |  |  |  |  |  |
| **Male** | 40 (47.6) | 49 (63.6) | 30 (38.0) | 60 (65.9) | 43 (47.8) | 41 (51.9) | 38 (49.4) | 46 (54.1) | 48 (55.8) | 40 (55.6) |
| **Gestation** | *n=76* | *n=74* | *n=79* | *n=90* | *n=90* | *n=76* | *n=76* | *n=85* | *n=86* | *n=72* |
| **Median(IQR^c^)** | 38 (34-38) | 38 (37-38) | 38 (37-38) | 38 (33-38) | 40 (36-40) | 39 (35-40) | 39 (33-40) | 39 (36-40) | 39 (34-40) | 39 (37-40) |
| **≥37 weeks** | 48 (63.2) | 57 (77.0) | 61 (77.2) | 55 (61.1) | 64 (71.1) | 51 (67.1) | 49 (64.5) | 60 (70.6) | 57 (66.3) | 54 (75.0) |
| **<37 - ≥34 weeks** | 13 (17.1) | 10 (13.5) | 6 (7.6) | 10 (11.1) | 12 (13.3) | 12 (15.8) | 7 (9.2) | 13 (15.3) | 9 (10.5) | 8 (11.1) |
| **<34 weeks** | 15 (19.7) | 7 (9.5) | 12 (15.2) | 25 (27.8) | 14 (15.6) | 13 (17.1) | 20 (26.3) | 12 (14.1) | 20 (23.2) | 10 (13.9) |
| **Birth Weight** | *n=77* | *n=74* | *n=79* | *n=91* | *n=90* | *n=77* | *n=77* | *n=85* | *n=86* | *n=72* |
| **Median(IQR)** | 2700 (1950-3150) | 2773 (2300-3220) | 2850 (2280-3200) | 2600 (1855-3035) | 2800 (2380-3040) | 2800 (2100-3250) | 2810 (2010-3160) | 2940 (2635-3285) | 2870 (2060-3220) | 2993 (2525-3253) |
| **≥2500 grams** | 46 (59.7) | 47 (63.5) | 56 (70.9) | 50 (55.0) | 64 (71.1) | 51 (66.2) | 48 (62.3) | 66 (77.7) | 55 (64.0) | 56 (77.8) |
| **1500-2499 grams** | 19 (24.7) | 22 (29.7) | 18 (22.8) | 26 (28.5) | 20 (22.2) | 20 (26.0) | 17 (22.1) | 9 (10.6) | 19 (22.1) | 8 (11.1) |
| **1000-1499 grams** | 11 (14.3) | 3 (4.1) | 5 (6.3) | 8 (8.8) | 4 (4.5) | 5 (6.5) | 7 (9.1) | 8 (9.4) | 7 (8.1) | 7 (9.7) |
| **≤999 grams** | 1 (1.3) | 2 (2.7) | 0 | 7 (7.7) | 2 (2.2) | 1 (1.3) | 5 (6.5) | 2 (2.3) | 5 (5.8) | 1 (1.4) |
| **Outcome** |  |  |  |  |  |  |  |  |  |  |
| **Discharged** | 73 (86.9) | 64 (83.1) | 66 (84.6) | 78 (85.7) | 72 (80.0) | 70 (92.1) | 62 (80.5) | 72 (84.7) | 71 (82.6) | 60 (83.3) |
| **Demised** | 11 (13.1) | 13 (16.9) | 12 (15.4) | 13 (14.3) | 18 (20.0) | 6 (7.9) | 15 (19.5) | 13 (15.3) | 15 (17.4) | 12 (16.7) |
| **Culture site** |  |  |  |  |  |  |  |  |  |  |
| **Blood only** | 53 (63.1) | 47 (61.0) | 44 (55.7) | 67 (73.6) | 53 (58.9) | 56 (70.9) | 56 (72.7) | 59 (69.4) | 56 (65.1) | 47 (65.3) |
| **CSF^d^ only** | 4 (4.8) | 11 (14.3) | 14 (17.7) | 7 (7.7) | 10 (11.1) | 6 (7.6) | 6 (7.8) | 5 (5.9) | 14 (16.3) | 6 (8.3) |
| **Blood and CSF** | 15 (17.9) | 11 (14.3) | 11 (13.9) | 9 (9.9) | 21 (23.3) | 15 (20.0) | 12 (15.6) | 16 (18.8) | 11 (12.8) | 12 (16.7) |
| **CSF suggestive^e^** | 12 (14.2) | 8 (10.4) | 10 (12.7) | 8 (8.8) | 6 (6.7) | 2 (2.5) | 3 (3.9) | 5 (5.9) | 5 (5.8) | 7 (9.7) |

^a^EOD- Early-onset disease, ^b^LOD- Late-onset disease, ^c^IQR- interquartile range, ^d^CSF- Cerebrospinal fluid, ^e^CSF suggestive of meningitis was defined as pleocytosis ≥20 cells/mm3 for <28 day-olds and ≥10 cells/mm3 for 29-89 day-olds with no adjustment made for traumatic taps and in the absence of positive CSF culture. *Data from 2005 to 2008 and from 2012 to 2013 have been previously reported.
