# Supplementary material for: Temporal Changes in Invasive Group B Streptococcus Serotypes: Implications for Vaccine Development
Source: PLoS One. 2016 Dec 30;11(12):e0169101. doi: 10.1371/journal.pone.0169101 (PMC5201280; doi:10.1371/journal.pone.0169101)
Supplement: S3 Table — aEOD- Early-onset disease, bLOD- Late-onset disease, crelative risk and 95% confidence interval comparing HIV-exposed and unexposed infants. (DOCX) [file pone.0169101.s005.docx]

S3 Table: Yearly incidence (95% Confidence interval) of infants with invasive Group B streptococcus disease

|  | **2005** | **2006** | **2007** | **2008** | **2009** | **2010** | **2011** | **2012** | **2013** | **2014** | **Overall** |
| --- | --- | --- | --- | --- | --- | --- | --- | --- | --- | --- | --- |
| **Overall** | 2.87  (2.29-3.56) | 2.46  (1.94-3.07) | 2.44  (1.93-3.04) | 2.84  (2.29-3.49) | 2.70  (2.17-3.31) | 2.44  (1.94-3.05) | 2.36  (1.86-2.95) | 2.71  (2.16-3.35) | 2.69  (2.16-3.33) | 2.39  (1.87-3.00) | 2.59  (2.42-2.77) |
| **EOD^a^** | 1.57  (1.15-2.10) | 1.28  (0.91-1.74) | 1.27  (0.91-1.72) | 1.69  (1.27-2.20) | 1.17  (0.83-1.60) | 1.21  (0.86-1.65) | 1.44  (1.06-1.91) | 1.75  (1.32-2.28) | 1.47  (1.08-1.96) | 1.29  (0.92-1.77) | 1.41  (1.28-1.55) |
| **LOD^b^** | 1.30  (0.92-1.78) | 1.18  (0.83-1.63) | 1.17  (0.19-1.61) | 1.16  (0.81-1.59) | 1.53  (1.14-2.01) | 1.24  (0.89-1.69) | 0.92  (0.62-1.31) | 0.96  (0.65-1.36) | 1.22  (0.87-1.07) | 1.09  (0.75-1.54) | 1.18  (1.06-1.30) |
|  |  |  |  |  |  |  |  |  |  |  |  |
| **HIV-exposed** |  |  |  |  |  |  |  |  |  |  |  |
| **All** | 3.80  (2.67-5.26) | 3.94  (2.79-5.40) | 2.53  (1.64-3.74) | 4.59  (3.34-6.16) | 4.33  (3.13-5.82) | 3.56  (2.48-4.95) | 3.63  (2.51-5.06) | 3.30  (2.25-4.68) | 3.50  (2.38-4.96) | 3.34  (2.24-4.79) | 3.66  (3.28-4.06) |
| **EOD** | 1.37  (0.73-2.35) | 1.87  (1.11-2.95) | 1.22  (0.63-2.12) | 2.30  (1.44-3.48) | 1.41  (0.77-2.36) | 1.43  (0.78-2.39) | 1.71  (0.98-2.77) | 1.70  (0.98-2.77) | 1.02  (0.46-1.93) | 1.04  (0.47-1.97) | 1.51  (1.27-1.78) |
| **LOD** | 2.43  (1.54-3.64) | 2.07  (1.27-3.20) | 1.32  (0.70-2.25) | 2.30  (1.44-3.48) | 2.92  (1.96-4.19) | 2.14  (1.32-3.27) | 1.92  (1.14-3.03) | 1.60  (0.90-2.63) | 2.48  (1.56-3.75) | 2.30  (1.41-3.55) | 2.15  (1.86-2.46) |
|  |  |  |  |  |  |  |  |  |  |  |  |
| **HIV-unexposed** |  |  |  |  |  |  |  |  |  |  |  |
| **All** | 1.92  (1.36-2.64) | 1.43  (0.98-2.03) | 1.91  (1.39-2.58) | 1.96  (1.42-2.63) | 1.84  (1.33-2.47) | 1.82  (1.31-2.47) | 1.55  (1.08-2.14) | 2.32  (1.73-3.05) | 2.34  (1.76-3.06) | 1.96  (1.41-2.64) | 1.90  (1.73-2.10) |
| **EOD** | 1.32  (0.86-1.93) | 0.88  (0.53-1.37) | 1.16  (0.76-1.69) | 1.29  (0.87-1.85) | 1.03  (0.66-1.53) | 1.02  (0.65-1.53) | 1.03  (0.66-1.53) | 1.73  (1.22-2.37) | 1.61  (1.13-2.21) | 1.40  (0.94-1.99) | 1.24  (1.10-1.40) |
| **LOD** | 0.61  (0.31-1.06) | 0.55  (0.29-0.97) | 0.76  (0.44-1.21) | 0.67  (0.37-1.10) | 0.81  (0.49-1.27) | 0.80  (0.47-1.27) | 0.52  (0.27-0.90) | 0.59  (0.32-1.01) | 0.74  (0.43-1.18) | 0.56  (0.29-0.98) | 0.66  (0.56-0.78) |
|  |  |  |  |  |  |  |  |  |  |  |  |
| **Relative risk^c^** |  |  |  |  |  |  |  |  |  |  |  |
| **Overall** | 1.98  (1.25-3.12) | 2.75  (1.71-4.42) | 1.32  (0.81-2.17) | 2.34  (1.55-3.56) | 2.36  (1.55-3.59) | 1.95  (1.25-3.07) | 2.35  (1.47-3.75) | 1.43  (0.91-2.23) | 1.49  (0.96-2.32) | 1.71  (1.07-2.74) | 1.92  (1.67-2.21) |
| **EOD** | 1.04  (0.55-2.03) | 2.13  (1.18-4.05) | 1.05  (0.53-2.08) | 1.78  (1.02-3.09) | 1.37  (0.71-2.66) | 1.39  (0.72-2.71) | 1.66  (0.88-3.12) | 0.99  (0.55-1.77) | 0.63  (0.31-1.31) | 0.74  (0.35-1.56) | 1.22  (0.99-1.49) |
| **LOD** | 4.00  (1.99-8.03) | 3.75  (1.83-7.66) | 1.74  (0.85-3.59) | 3.44  (1.79-6.63) | 3.60  (2.02-6.41) | 2.67  (1.42-5.01) | 3.73  (1.80-7.73) | 2.71  (1.29-5.68) | 3.37  (1.79-6.33) | 4.12  (2.02-8.43) | 3.24  (2.62-4.01) |

^a^EOD- Early-onset disease, ^b^LOD- Late-onset disease, ^c^relative risk and 95% confidence interval comparing HIV-exposed and unexposed infants.
