# Supplementary material for: Temporal Changes in Invasive Group B Streptococcus Serotypes: Implications for Vaccine Development
Source: PLoS One. 2016 Dec 30;11(12):e0169101. doi: 10.1371/journal.pone.0169101 (PMC5201280; doi:10.1371/journal.pone.0169101)
Supplement: S4 Table — Serotype data was missing on 47 (56.0%) in 2005, 27 (35.1%) in 2006, 27 (34.2%) in 2007, 21 (23.1%) in 2008, 27 (30.0%) in 2009, 13 (16.5%) in 2010, 13 (16.9%) in 2011, 8 (9.4%) in 2012, 2 (2.3%) in 2013 and nil for 2014. (DOCX) [file pone.0169101.s006.docx]

S4 Table: Serotype proportion (95% confidence interval) of infants with invasive GBS disease

| **Serotype** | **2005** | **2006** | **2007** | **2008** | **2009** | **2010** | **2011** | **2012** | **2013** | **2014** | **Total** | **EOD** | **LOD** |
| --- | --- | --- | --- | --- | --- | --- | --- | --- | --- | --- | --- | --- | --- |
| **Ia** | 16.2  (6.2-32.0)  n=6 | 24.0  (13.1-38.2)  n=12 | 9.6  (3.2-21.0) n=5 | 25.7  (16.0-37.6) n=18 | 25.4  (15.3-37.9) n=16 | 19.7  (10.9-31.3) n=13 | 32.8  (21.6-45.7) n=21 | 44.2  (32.8-55.9) n=34 | 42.9  (32.1-54.1) n=36 | 25.0  (15.5-36.6) n=18 | **28.2**  **(24.7-31.9) n=179** | **34.7**  **(29.8-39.9) n=125** | **19.6**  **(15.1-24.8) n=54** |
| **Ib** | - | 2.0  (0.1-10.5) n=1 | 9.6  (3.2-21.0) n=5 | 5.7  (1.6-13.9) n=4 | - | - | 3.1  (0.4-10.8) n=2 | 7.8  (2.9-16.2) n=6 | 2.4  (0.3-8.3) n=2 | 2.8  (0.3-9.7) n=2 | **3.5**  **(2.2-5.2) n=22** | **4.7**  **(2.8-7.5) n=17** | **1.8**  **(0.6-4.2)**  **n=5** |
| **II** | 2.7  (0.7-14.2)  n=1 | 2.0  (0.1-10.5) n=1 | 3.9  (0.5-13.2) n=2 | 2.9  (0.4-9.9) n=2 | 3.2  (0.4-11.0) n=2 | 6.1  (1.7-14.8) n=4 | 3.1  (0.4-10.8) n=2 | 1.3  (0.1-7.0) n=1 | 2.4  (0.3-8.3) n=2 | 8.3  (3.1-17.2) n=6 | **3.6**  **(2.3-5.4) n=23** | **5.6**  **(3.4-8.5) n=20** | **1.1**  **(0.2-3.2)**  **n=3** |
| **III** | 70.3  (53.0-84.1)  n=26 | 66.0  (51.2-78.8) n=33 | 73.1  (59.0-84.4) n=38 | 54.3  (41.9-66.3) n=38 | 65.1  (52.0-76.7) n=41 | 68.1  (55.6-79.1) n=45 | 50.0  (37.2-62.8) n=32 | 41.5  (30.4-53.4) n=32 | 39.3  (28.8-50.6) n=33 | 48.6  (36.7-60.7) n=35 | **55.6**  **(51.6-59.5) n=353** | **41.7**  **(36.5-47.0) n=150** | **73.8**  **(68.2-78.9) n=203** |
| **IV** | 2.7  (0.7-14.2)  n=1 | - | 1.9  (0.1-10.3) n=1 | 4.3  (0.9-12.0) n=3 | - | - | - | - | 1.2  (0.1-6.5) n=1 | 1.4  (0.1-7.5) n=1 | **1.1**  **(0.4-2.3)**  **n=7** | **1.9**  **(0.8-4.0)**  **n=7** | **-** |
| **V** | 8.1  (1.7-21.9) n=3 | 6.0  (1.3-16.6) n=3 | 1.9  (0.1-10.3) n=1 | 7.1  (2.4-15.9) n=5 | 6.3  (1.8-15.5) n=4 | 6.1  (1.7-14.8) n=4 | 9.4  (3.5-19.3) n=6 | 5.2  (1.4-12.7) n=4 | 11.9  (5.9-20.8) n=10 | 13.9  (6.8-24.1) n=10 | **7.9**  **(5.9-10.3) n=50** | **11.4**  **(8.3-15.1) n=41** | **3.3**  **(1.5-6.1)**  **n=9** |
| **VI** | - | - | - | - | - | - | 1.6  (0.1-8.4) n=1 | - | - | - | **0.2**  **(0.1-0.8)**  **n=1** | **-** | **0.4**  **(0.1-2.0)**  **n=1** |
| **Total** | **37** | **50** | **52** | **70** | **63** | **66** | **64** | **77** | **84** | **72** | **635** | **360** | **275** |

Serotype data was missing on 47 (56.0%) in 2005, 27 (35.1%) in 2006, 27 (34.2%) in 2007, 21 (23.1%) in 2008, 27 (30.0%) in 2009, 13 (16.5%) in 2010, 13 (16.9%) in 2011, 8 (9.4%) in 2012, 2 (2.3%) in 2013 and nil for 2014.
